# Reduced Feeding Frequency Improves Feed Efficiency Associated With Altered Fecal Microbiota and Bile Acid Composition in Pigs

**DOI:** 10.3389/fmicb.2021.761210

**Published:** 2021-10-12

**Authors:** Honglin Yan, Wenzhuo Wei, Luga Hu, Yong Zhang, Hongfu Zhang, Jingbo Liu

**Affiliations:** ^1^School of Life Science and Engineering, Southwest University of Science and Technology, Mianyang, China; ^2^State Key Laboratory of Animal Nutrition, Institute of Animal Sciences, Chinese Academy of Agricultural Sciences, Beijing, China

**Keywords:** bi-phasic feeding, feeding frequency, feed efficiency, microbiome, metabolome, bile acids, pigs

## Abstract

A biphasic feeding regimen exerts an improvement effect on feed efficiency of pigs. While gut microbiome and metabolome are known to affect the host phenotype, so far the effects of reduced feeding frequency on fecal microbiota and their metabolism in pigs remain unclear. Here, the combination of 16S rRNA sequencing technique as well as untargeted and targeted metabolome analyses was adopted to investigate the fecal microbiome and metabolome of growing–finishing pigs in response to a biphasic feeding [two meals per day (M2)] pattern. Sixty crossbred barrows were randomly assigned into two groups with 10 replicates (three pigs/pen), namely, the free-access feeding group (FA) and the M2 group. Pigs in the FA group were fed free access while those in the M2 group were fed *ad libitum* twice daily for 1 h at 8:00 and 18:00. Results showed that pigs fed biphasically exhibited increased feed efficiency compared to FA pigs. The Shannon and Simpson indexes were significantly increased by reducing the feeding frequency. In the biphasic-fed pigs, the relative abundances of *Subdoligranulum*, *Roseburia*, *Mitsuokella*, and *Terrisporobacter* were significantly increased while the relative abundances of *unidentified_Spirochaetaceae*, *Methanobrevibacter*, *unidentified_Bacteroidales*, *Alloprevotella*, *Parabacteroides*, and *Bacteroides* were significantly decreased compared to FA pigs. Partial least-square discriminant analysis (PLS-DA) analysis revealed an obvious variation between the FA and M2 groups; the differential features were mainly involved in arginine, proline, glycine, serine, threonine, and tryptophan metabolism as well as primary bile acid (BA) biosynthesis. In addition, the changes in the microbial genera were correlated with the differential fecal metabolites. A biphasic feeding regimen significantly increased the abundances of primary BAs and secondary BAs in feces of pigs, and the differentially enriched BAs were positively correlated with some specific genera. Taken together, these results suggest that the improvement effect of a reduced feeding frequency on feed efficiency of pigs might be associated with the altered fecal microbial composition and fecal metabolite profile in particular the enlarged stool BA pool.

## Introduction

Feeding frequency has been suggested to be an important factor in energy balance regulation and weight management in rodents and humans (Paoli et al., 2019). Likewise, feeding frequency has been regarded as a critical factor that affects weight and carcass composition in pigs (Le Naou et al., 2014). Some studies have demonstrated that reducing meal frequency impaired the feed efficiency, while other studies contend that reduced meal frequency elevated the feed efficiency of pigs (Schneider et al., 2011; Colpoys et al., 2016; Liu et al., 2017). A previous study has shown that the duration of each feeding bout is critical for the improvement effect of reduced meal frequency on feed conversion efficiency (Newman et al., 2014). After the comparison of the feeding regimen design between previous studies, it can be summarized that pigs fed for two 60-min intervals exhibited improved feed efficiency compared to those fed for a higher meal frequency or *ad libitum* (Le Naou et al., 2014; Newman et al., 2014; Liu et al., 2017). Under the condition of reduced feeding frequency, the improved feed efficiency might be attributed to the changes in energy partitioning or the form of energy storage (Van Milgen, 2002; Le Naou et al., 2014). Consistently, our previous study showed that reduced meal frequency improved the feed efficiency and decreased the lipid deposition of pigs (Yan et al., 2020). Therefore, reduced meal frequency might exert a positive effect on feed utilization efficiency of pigs, but the underlying mechanisms remain unknown.

Gut microbes have been regarded as a critical factor in the regulation of host metabolism in particular to lipogenesis (Bäckhed et al., 2004). Indeed, fecal microbiota transplantation can transfer obesity-associated phenotypes from donors to recipients (Duca et al., 2014). The conversion of carbohydrate to lipid has been shown to be less energetically favored than storage as glycogen (Van Milgen, 2002). Thus, gut microbiota could be an environmental factor affecting the conversion of feed into weight gain. A previous study demonstrated that pigs clustered into the *Prevotella* enterotype tended to have a lower feed efficiency than those pigs clustered into the *Treponema* enterotype (Yang et al., 2017). Additionally, transplanting fecal microbiota from highly feed-efficient pigs into pregnant sows has resulted in better feed efficiency in offspring (McCormack et al., 2019). The gut microbiota have great potential to produce bioactive compounds that may signal the host by activating cognate receptors in various cells (Holmes et al., 2012). One important class of microbial metabolites, bile acids (BAs), is produced in the liver from cholesterol and chemically modified in the intestine by gut microbiota (Wahlström et al., 2016). BAs play a critical role in the emulsification of dietary lipids and also act as signaling molecules in several host processes (Swann et al., 2011). Several studies have demonstrated that the alterations in gut microbiota would elicit the changes in BA profiles, which in turn impact host digestive and signaling processes (Wahlström et al., 2016; Sun et al., 2019). The modifications in the proportion of BAs in specific tissues have been associated with altered whole-body energy expenditure and body composition (Joyce et al., 2014). Indeed, mice abundant in muricholic BAs showed resistance to diet-induced weight gain (Bonde et al., 2016). Likewise, the antimicrobial promotion of pig growth has been shown to be associated with the remodeling of the BA signature in the intestine (Ipharraguerre et al., 2018).

The feeding pattern has been shown to significantly affect the gut microbiota composition in mice. In addition, time-restricted feeding increased the concentrations of primary BAs in feces of mice (Zarrinpar et al., 2014). This suggests that the gut microbiota and its metabolite profile are modified in response to altered feeding patterns. However, it is unclear whether the gut microbiota and metabolism are shaped by feeding frequency, and if so whether these alterations play a role in affecting host growth traits. Hence, we sought to determine whether the effects of feeding frequency on growth performance of pigs are associated with the microbiome and metabolome profiles.

## Materials and Methods

This experiment was approved by the Institutional Animal Care and Use Committee of Southwest University of Science and Technology (Reference number: L2020005).

### Animals, Experimental Design, and Diets

A total of 60 growing barrows (Duroc × Landrace × Yorkshire), with an average weight of 28.03 kg, were allotted to one of the two feeding schedules [two meals per day (M2) or free access to the diet (FA)] in a randomized complete block design with body weight (BW) as blocking criterion. Each treatment contained 10 pens with 3 pigs per pen. Pigs were fed the same corn–soybean meal-based diet (Supplementary Table 1) which was offered in pellet form. The diet was formulated to meet or exceed the nutrient requirements for pigs at the corresponding growth stage as recommended by the National Research Council (2012). Pigs in the M2 group received their diet at 8:00 a.m. and 6:00 p.m. every day with free access to diet during feeding. Each meal lasted for 1 h, and the feed residues were removed from the feeder and weighed at 9:00 a.m. and 7:00 p.m. every day. Pigs in the FA group were allowed to ingest feed on an *ad libitum* basis every 24 h. Regardless of the dietary treatment, all pigs had free access to water. Pigs were housed in an environmentally controlled room with a temperature in the range of 18 to 22°C during the 16-week experimental period.

### Growth Traits Measurement and Sample Collection

The initial BW of the barrows was measured on the morning of day 1 of the experiment after overnight fasting. The fasted BW was determined on the last day of the trial to calculate the average daily gain (ADG) during the whole trial. The average daily feed intake (ADFI) was measured based on the amount of consumed feed during the whole experimental period. The ratio of feed to gain for the whole experimental period was then calculated. On the last day of the experiment, for each pen, the spontaneously excreted feces from three pigs were collected and mixed to one pooled sample which was then stored at −80°C, pending the isolation of genomic DNA as well as analysis for metabolome and BA profiles.

### Genomic DNA Extraction and Sequencing

For each feces sample, the DNA was extracted using the QIAamp Fast DNA Stool Mini Kits (Qiagen, Beijing, China) according to the manufacturer’s instructions. The quantity and integrity of isolated DNA were determined on a NanoDrop ND-1000 instrument and evaluated visually by agarose gel electrophoresis, respectively. During DNA extraction, a negative control sample, sterilized water, was included and showed no detectable PCR product. The V4 region of the bacterial 16S rRNA gene was amplified using common primers (515F and 806R). The PCR products were pooled and purified with Agencourt AMPure XP beads (Beckman Coulter, Brea, CA, United States) and the MinElute PCR Purification Kit (Qiagen, Beijing, China). These purified pooled amplicons were used to construct Illumina libraries by using the Ovation Rapid DR Multiplex System 1-96 (NuGEN, San Carlos, CA, United States). All sample libraries were sent to Novogene Co., Ltd., (Beijing, China) for 16S rRNA sequencing on the Illumina MiSeq platform using a PE250 sequencing strategy. The raw sequencing data can be reached with the accession number PRJNA756094 in the NCBI BioProject database.

### Fecal Microbiota Analysis

The raw Illumina data were processed with the Mothur software (v1.3.6). After removing the primer and barcode sequences, and the low-quality reads, paired-end clean sequences were assembled into tags with the overlapping relationship. To minimize the effect of the sequencing depth on the determination of microbial composition, the library size of each sample was rarefied to 29,915 tag-depth. Tags were clustered into OTUs based on 97% similarity by USEARCH v7.0.1001. The representative sequence of each OTU cluster was picked and used for taxonomic classification against the Ribosomal Database Project database with RDP v2.6. The resulting OTU abundance table and the OTU taxonomic assignment table from Mothur software were used to calculate the alpha diversity indexes of communities by R studio v3.4.1. The beta diversity was performed to compare the structural difference of the microbiota communities across samples using Bray–Curtis distance and visualized by non-metric multidimensional scaling (NMDS) analysis.

### Metabolomics Analysis

Fecal samples were thawed on ice, and metabolites were extracted with 50% methanol buffer, as previously described (Zheng et al., 2021). LC--MS/MS analysis was performed on an ultra-high-performance liquid chromatography (UHPLC) system (Agilent Technologies, Santa Clara, CA, United States) coupled to a high-resolution mass spectrometer TripleTOF 6600, which was operated in both positive and negative ion modes. The QC sample was obtained by mixing a small and equal volume of each experimental sample and injected at regular intervals in order to monitor the stability of the analysis. The raw data files were converted into mzXML format and then processed by the XCMS for peak detection, alignment, and data filtering. The metabolites were identified by comparison with the internal library using the mass-to-charge ratio (*m/z*), retention time, and chromatographic data. Supervised partial least-square discriminant analysis (PLS-DA) was performed using the MetaboAnalyst 5.0^1^ web-based system. The metabolites with VIP > 1, *p*-value < 0.05, and fold change (FC) ≥ 2 or FC ≤ 0.5 were considered significantly different. The significantly differentially abundant metabolites screened from untargeted metabolomics were imported into the MetaboAnalyst 5.0 database to perform pathway analysis.

### Quantification of Bile Acids in Feces

The BAs in feces were extracted according to the methods described previously (Fang et al., 2018). Briefly, 50 to 80 mg of lyophilized feces was added into a mixture of pre-cold sodium acetate buffer (50 mM, pH 5.6) and ethanol (chromatography grade), followed by 2-min mixing and 20-min centrifugation at 20,000 *g*. The supernatant was diluted five times with a sodium acetate buffer and applied to a Bond Elut C18 cartridge (Varian, Palo Alto, CA, United States), which was then washed with 25% ethanol. BAs were eluted with 5 ml methanol. After the solvent was evaporated with nitrogen gas, the residue was dissolved in 1 ml of methanol and then passed through a 0.45-μm Milled-LG filter (Millipore, Billerica, MA, United States) to get the filtrate for BA analyses. A Waters Xevo TQ-S LC/MS mass spectrometer (Waters, Milford, MA, United States) equipped with an ESI source was adopted to determine the BA profile in samples, and all the assay conditions were in accordance with a previous study (Fang et al., 2018). The concentration of each BA in samples was determined based on the series dilutions of standards, and good linearity was confirmed. In this study, a total of 18 BA standards were purchased from Sigma-Aldrich (Merck KGaA, Darmstadt, Germany).

### Statistical Analysis

The Student *t*-test was used to analyze the data on growth performance, BA profile, and bacterial alpha-diversity indexes as well as the metabolites identified from metabolomics. The Wilcoxon rank-sum test was used to compare differentially abundant bacteria between two groups. The Spearman’s correlation between the differentially abundant metabolites screened by metabolomics and the significantly different genera obtained by 16S rDNA sequencing analysis as well as the Spearman’s correlation between top 30 genera and BAs were calculated by the ggcor R package. The multiple test corrections have been performed by using the Benjamini–Hochberg method, and an FDR-adjusted *p*-value less than 0.05 was considered as statistically significant.

## Results

### Feeding Pattern Affected Growth Performance of Pigs

Although no difference in final BW, average daily weight gain, and ADFI were observed between pigs fed *ad libitum* and pigs fed twice per day, the feed-to-gain ratio during the 16-week period was significantly decreased in pigs fed two meals per day compared to those which had free access to feed (Table 1, *p* < 0.05).

**TABLE 1:** Effects of feeding pattern on growth performance in growing–finishing pigs.

**Item**	**FA**	**M2**	***p*-values**
Initial body weight (kg)	28.73 ± 1.35	29.10 ± 1.08	0.831
Final body weight (kg)	122.58 ± 3.30	121.27 ± 3.40	0.786
Average daily weight gain (kg)	0.84 ± 0.02	0.85 ± 0.04	0.830
Average daily feed intake (kg)	2.38 ± 0.09	2.17 ± 0.09	0.112
Feed to gain ratio	2.84 ± 0.05	2.58 ± 0.09	0.026

*Data are means ± SE; *n* = 10 for each group. FA, pigs had free access to feed; M2, pigs were given two meals per day (0800 and 1800 h); each meal lasted 60 min.*

### Feeding Pattern Altered the Fecal Microbial Profiles of Pigs

**FIGURE 1:**
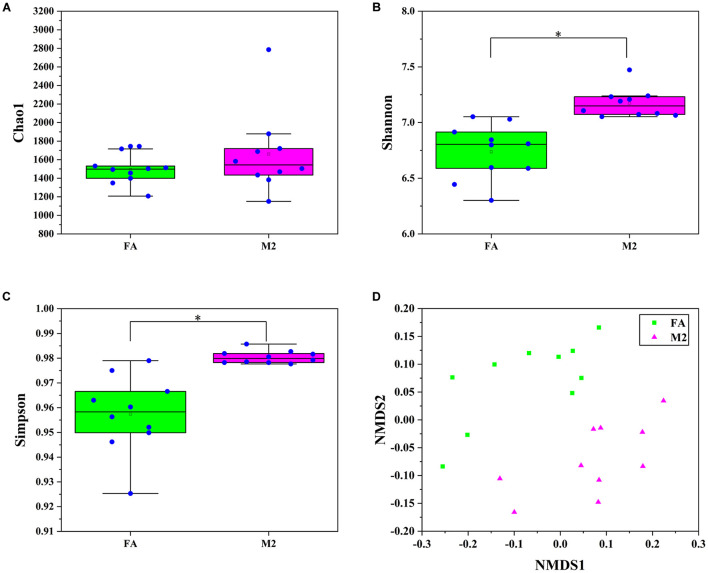
Fecal microbiome diversity and structure analysis. **(A)** Chao 1 index. **(B)** Shannon index. **(C)** Simpson index. **(D)** Non-metric multidimensional scaling (NMDS) ordination plot derived from Bray–Curtis distances in feces of FA and M2 pigs. FA, pigs had free access to feed; M2, pigs were given two meals per day (0800 and 1800 h); each meal lasted 60 min. ^∗^*p* < 0.05.

**FIGURE 2:**
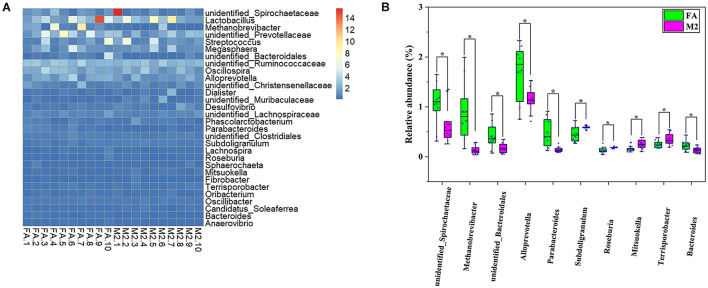
Generic differences between FA and M2 groups. **(A)** Heat map of the relative abundance of top 30 genera. **(B)** Significantly different genera in feces between FA and M2 groups. FA, pigs had free access to feed; M2, pigs were given two meals per day (0800 and 1800 h); each meal lasted 60 min. *p < 0.05.

A total of 20 pooled feces samples, which were obtained by mixing feces from three pigs in each pen, were used to determine the gut microbiota composition by 16S rRNA sequencing. The number of high-quality tags in all samples ranged from 29,915 to 56,560, which were normalized to 29,915 and clustered into 1642 OTUs at a 97% similarity cutoff. According to the diversity indices presented in Figure 1, the fecal microbiome of pigs in the M2 group exhibited the greatest diversity. Although no difference in Chao1 index between groups was observed (Figure 1A, *p* > 0.05), the Shannon and Simpson indexes of the feces samples from M2 pigs were higher than those from FA pigs (Figures 1B,C, *p* < 0.05). The similarity of microbiota compositions between samples was assessed by Bray–Curtis distance-based NMDS analysis. The NMDS-based map showed distinct differences between pigs fed twice per day and those fed *ad libitum* (Figure 1D, *p* < 0.05). Next, the bacterial communities were assessed by using heat maps to display dominant taxa at both the phylum (Supplementary Figure 1A) and genus levels (Figure 2A). The most dominant taxa at the phylum level in feces of FA and M2 pigs was *Firmicutes*, followed by *Bacteroidetes*, *Spirochaetes*, and *Euryarchaeota* or *Proteobacteria* (Supplementary Figure 1A and Supplementary Table 2). Phyla *Spirochaetes* and *Euryarchaeota* were found to be more abundant in feces of FA pigs compared to M2 pigs (Supplementary Figure 1B, *p* < 0.05). Reduced feeding frequency tended to decrease the relative abundance of *Melainabacteria* in feces of pigs (Supplementary Figure 1B, *p* < 0.05). At the genus level, there were seven dominant taxa (average abundance more than 1%) in both FA and M2 groups (Supplementary Table 3). At the genus level, *unidentified_Prevotellaceae* was the most predominant genus in feces of FA pigs, while *Lactobacillus* was the most abundant genus in feces of M2 pigs (Figure 2A and Supplementary Table 3). The relative abundances of *unidentified_Spirochaetaceae*, *Methanobrevibacter*, *unidentified_Bacteroidales*, *Alloprevotella*, *Parabacteroides*, and *Bacteroides* were significantly higher in feces of FA pigs compared to their counterparts which were fed twice per day. Reduced feeding frequency significantly increased the relative abundances of *Subdoligranulum*, *Roseburia*, *Mitsuokella*, and *Terrisporobacter* in feces of pigs (Figure 2B, *p* < 0.05).

### Feeding Pattern Changed Enrichment in Fecal Metabolic Pathways of Pigs

**FIGURE 3:**
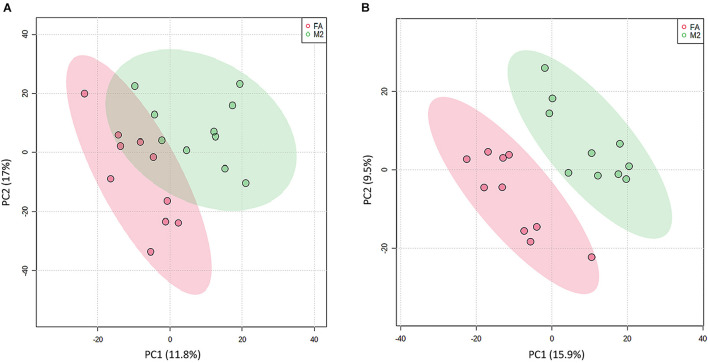
Partial least-square discriminant analysis plot based on fecal metabolites. PLS-DA plot of fecal metabolites detected in positive **(A)** and negative modes **(B)** between FA and M2 groups. FA, pigs had free access to feed; M2, pigs were given two meals per day (0800 and 1800 h); each meal lasted 60 min.

**FIGURE 4:**
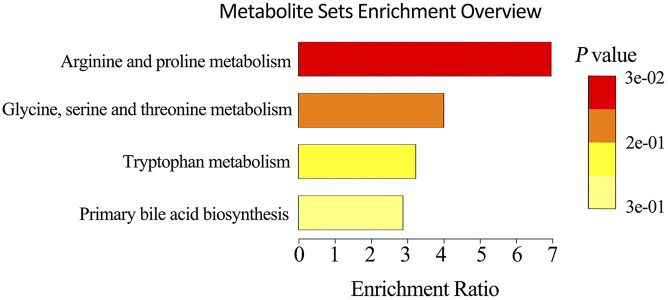
Metabolic pathway enrichment analysis. Overview of metabolites that were changed in the feces of M2 pigs compared to FA pigs. FA, pigs had free access to feed; M2, pigs were given two meals per day (0800 and 1800 h); each meal lasted 60 min.

To explore the metabolic changes occurring as a response to reduced feeding frequency, the LC–MS-based untargeted metabolomics was adopted to analyze the metabolite composition in feces of pigs. A PLS-DA was used to identify the altered metabolites in feces of M2 pigs compared to FA pigs. The PLS-DA model revealed metabolic profile differences between FA and M2 pigs (Figures 3A,B). A total of 12 differently enriched metabolites between FA and M2 pigs have been identified based on relative abundances and VIP values, of which 10 metabolites were upregulated and 2 metabolites were downregulated in M2 pigs. The largest upregulated metabolite in M2 pigs compared to FA pigs was chenodeoxycholic acid (Table 2). These differently enriched metabolites were mainly involved in arginine, proline, glycine, serine, threonine, and tryptophan metabolism as well as primary BA biosynthesis (Figure 4).

**TABLE 2:** Significantly different metabolites in feces between FA and M2 groups.

**Metabolites**	**FC (M2/FA)**	***p*-Value**	**VIP**	**Regulated**	**HMD**	**KEGG**
Chenodeoxycholic acid	5.8914	0.0200	1.97	Up	HMDB0000518	C02528
Chenodeoxycholic acid 3-sulfate	4.9306	0.0326	1.09	Up	HMDB0002586	NA
3,4-Hexahydroxydiphenoylarabinose	4.9230	0.0210	1.96	Up	HMDB0034113	NA
3-Methylindole	4.6007	0.0354	1.65	Up	HMDB0000466	C08313
Indoleacetic acid	4.6017	0.0352	1.81	Up	HMDB0000197	C00954
5-Amino-4-imidazole carboxylate	4.3272	0.0128	2.09	Up	METPA0605	C05516
Guanidineacetic acid	3.2773	0.0148	1.83	Up	HMDB0000128	C00581
4-Hydroxy-L-glutamic acid	2.8730	0.0067	2.24	Up	HMDB0002273	C03079
Guanidinosuccinic acid	2.0897	0.0089	1.85	Up	HMDB0003157	C03139
2-(7′-Methylthio) heptylmalic acid	0.2996	0.0155	1.66	Down	METPA1771	C17230
4-Hydroxylamino-2,6-dinitrotoluene	0.3615	0.0207	1.58	Down	METPA1824	C16392

*FC, fold change; FA, pigs had free access to feed; M2, pigs were given two meals per day (0800 and 1800 h); each meal lasted 60 min. The metabolites with significant difference (multidimensional statistical analysis of VIP > 1 and univariate statistical analysis of *p*-value < 0.05) and fold change (FC) ≥ 2 or FC ≤ 0.5 were listed.*

### Correlation of Fecal Microbiota With Fecal Metabolic Phenotype

The correlation analyses between the altered genus and differently enriched metabolites, which were identified by 16S rRNA sequencing and LC–MS-based untargeted metabolomics, respectively, were performed and visualized in a heat map (Supplementary Figure 2). Genus *Subdoligranulum* was positively correlated with guanidinosuccinic acid, 3,4-hexahydroxydiphenoylarabinose, 5-amino-4-imidazole carboxylate, 3-methyl indole, 4-hydroxy-L-glutamic acid, chenodeoxycholic acid, and chenodeoxycholic acid 3-sulfate and negatively correlated with 2-(7′-methylthio)heptylmalic acid (*p* < 0.05). Genus *Roseburia* was positively correlated with guanidineacetic acid, chenodeoxycholic acid, and chenodeoxycholic acid 3-sulfate (*p* < 0.05). Genus Terrisporobacter was positively correlated with guanidineacetic acid, 5-amino-4-imidazole carboxylate, indoleacetic acid, 4-hydroxy-L-glutamic acid, and chenodeoxycholic acid (*p* < 0.05). Genus Methanobrevibacter was positively correlated with 2-(7′-methylthio) heptylmalic acid and negatively correlated with guanidineacetic acid, 3-methyl indole, and 4-hydroxy-L-glutamic acid (*p* < 0.05).

### Feeding Pattern-Shaped Bile Acid Profile in Feces of Pigs

**FIGURE 5:**
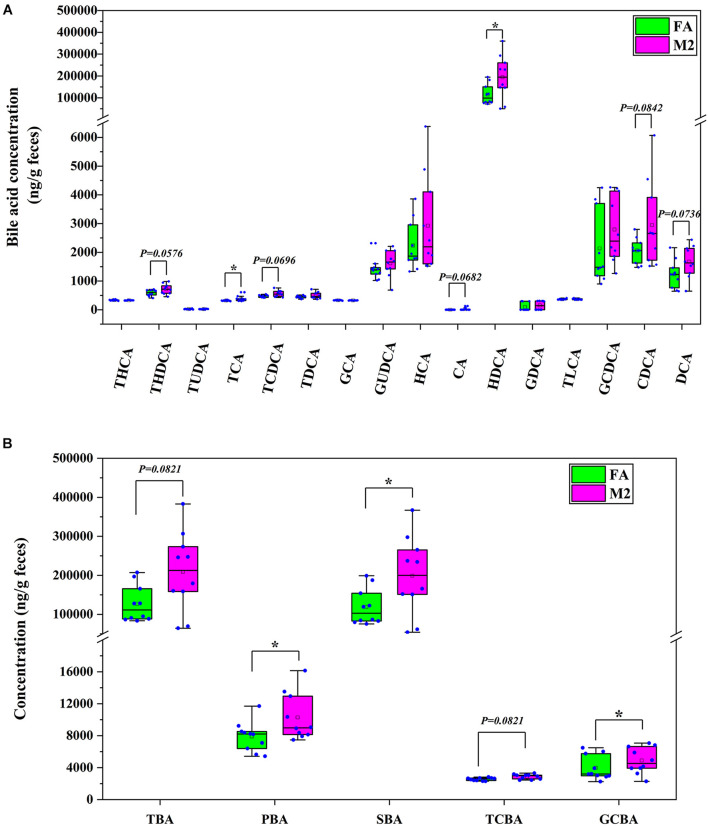
Bile acid profile in feces of pigs. **(A)** The concentration of each bile acid in feces. **(B)** The concentration of each type of bile acid in feces. FA, pigs had free access to feed; M2, pigs were given two meals per day (0800 and 1800 h); each meal lasted 60 min. *p < 0.05. THCA, tauro-hyocholic acid; THDCA, tauro-hyodeoxycholic acid; TUDCA, tauro-ursodeoxycholic acid; TCA, tauro-cholic acid; TCDCA, tauro-chenodeoxycholic acid; TDCA, tauro-deoxycholic acid; GCA, glycol-cholic acid; GUDCA, glycol-ursodeoxycholic acid; HCA, hyocholic acid; CA, cholic acid; HDCA, hyodeoxycholic acid; GDCA, glycol-deoxycholic acid; TLCA, tauro-lithocholic acid; GCDCA, glycol-chenodeoxycholic acid; CDCA, chenodeoxycholic acid; DCA, deoxycholic acid; TBA, total bile acid; PBA, primary bile acid; SBA, secondary bile acid; TCBA, taurine-conjugated bile acid; GCBA, glycine-conjugated bile acid.

Based on the alterations in fecal metabolome in response to reduced feeding frequency, a further investigation of BA concentrations in feces was performed and is presented in Figure 5. The concentrations of tauro-cholic acid and hyodeoxycholic acid were higher in feces of M2 pigs compared to FA pigs (Figure 5A, *p* < 0.05). Reduced feeding frequency tended to increase the abundances of tauro-hyodeoxycholic acid (*p* = 0.0576), tauro-chenodeoxycholic acid (*p* = 0.0696), cholic acid (*p* = 0.0682), chenodeoxycholic acid (*p* = 0.0842), and deoxycholic acid (*p* = 0.0736) in feces of pigs (Figure 5A). Analogously, the concentrations of primary BA, secondary BA, and glycine-conjugated BA were significantly higher in feces of M2 pigs than that of FA pigs (Figure 5B, *p* < 0.05). There was a tendency toward a higher concentration of total BA in M2 pigs compared to FA pigs (Figure 5B, *p* = 0.0821). Reduced feeding frequency tended to increase the abundance of taurine-conjugated BA in feces of pigs (Figure 5B, *p* = 0.0821).

### The Correlation Between Fecal Microbiota and Fecal Bile Acid Profile

**FIGURE 6:**
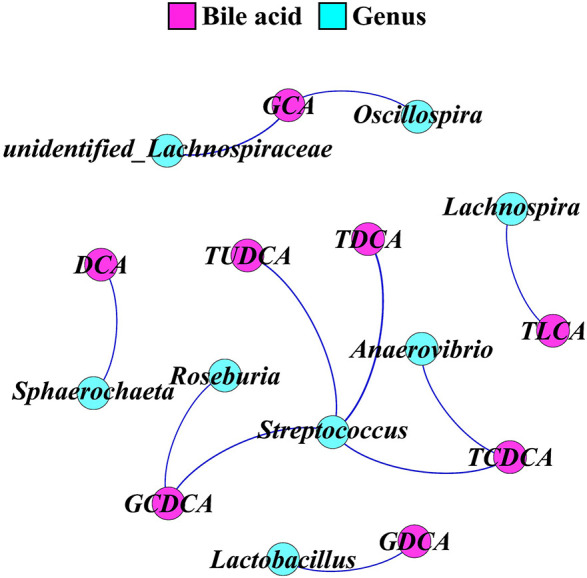
A network for correlation analysis between the top 30 genera and fecal bile acids using relevant networking. The nodes were colored according to bile acid and genus. Only correlations with Spearman’s coefficient *r* > 0.5 and *p* < 0.05 are shown. TUDCA, tauro-ursodeoxycholic acid; TCDCA, tauro-chenodeoxycholic acid; TDCA, tauro-deoxycholic acid; GCA, glycol-cholic acid; GDCA, glycol-deoxycholic acid; TLCA, tauro-lithocholic acid; GCDCA, glycol-chenodeoxycholic acid; DCA, deoxycholic acid.

Network regulation analysis results of BAs and top 30 genera in feces are shown in Figure 6. Genus *Streptococcus* was significantly positively correlated with glycol-chenodeoxycholic acid, tauro-deoxycholic acid, tauro-ursodeoxycholic acid, and tauro-chenodeoxycholic acid (*p* < 0.05). Genus *Roseburia* was significantly positively correlated with glycol-chenodeoxycholic acid (*p* < 0.05). Genus *Anaerovibrio* was significantly positively correlated with tauro-chenodeoxycholic acid (*p* < 0.05). Genus *Lactobacillus* was significantly positively correlated with glycol-deoxycholic acid (*p* < 0.05). Genera *Lachnospira* and *Sphaerochaeta* were significantly positively correlated with tauro-lithocholic acid and deoxycholic acid, respectively (*p* < 0.05). Genus *unidentified_Lachnospiraceae* and *Oscillospira* were significantly positively correlated with glycol-cholic acid (*p* < 0.05).

## Discussion

The influence of feeding frequency on the growth performance of pigs has been a topic of interest for many years. However, it is inconclusive whether higher or lower meal frequency is associated with higher feed conversion efficiency. Both earlier and more recent studies have drawn contradictory conclusions about the effects of biphasic feeding on pig growth performance (Allee et al., 1972; Romsos et al., 1978; Le Naou et al., 2014; Colpoys et al., 2016). A study conducted by Newman et al. (2014) showed that the lasting time of each meal determined the outcome of feeding frequency on feed conversion efficiency. Growing pigs fed biphasically for two 60-min periods rather than those fed for two 90-min meals exhibited a higher feed conversion rate compared to pigs fed *ad libitum* (Newman et al., 2014). A previous study also confirmed that the feed conversion efficiency of young pigs that had access to feed during 1 h at two time points was higher than those fed 12 meals per day (Le Naou et al., 2014). Consistently, in the present study, pigs in the M2 group that were fed for two 60-min meals had improved feed conversion efficiency than their counterparts that had free access to feed. Therefore, under the condition of restricted feeding frequency, its improved effects on growth performance of pigs in particular to feed conversion efficiency might largely depend on the duration of each meal.

Previous studies have shown that the biphasic feeding regimen improved the feed efficiency of pigs which were associated with altered energy partitioning and storage form (protein or lipid), as indicated by higher lean tissue content and/or decreased fat deposition in biphasic-fed pigs (Newman et al., 2014; Liu et al., 2017; Yan et al., 2020). Gut microbiota have been recognized as a critical factor influencing the energy partitioning between lean and fat tissues of the host (Bäckhed et al., 2004). The important role of gut microbiota in determining muscle growth and the myofiber characteristics of pigs and rodents has been revealed by using the germ-free mouse model (Yan et al., 2016; Lahiri et al., 2019). Gut microbiota have also been shown to regulate the fat deposition of chickens largely independent of host genetics (Wen et al., 2019). Altogether, it can be speculated that the changes in gut microbiota composition could be responsible for the improvement effect of biphasic feeding pattern on feed conversion efficiency in pigs. In the present study, the gut microbiota composition of pigs was shifted by the biphasic feeding regimen. Feeding pigs biphasically significantly elevated the Shannon and Simpson indexes, indicating a more diverse gut microbiome in M2 pigs than FA pigs. Biphasic-fed pigs also exhibited a higher feed efficiency, which is consistent with previous findings that high feed efficiency crossbred pigs had a higher Shannon index (Quan et al., 2018). Previous studies also indicated that low diversity in a microbiome could be a good predictor of a poor health status and lower feed efficiency (Bergamaschi et al., 2020a,b). *Firmicutes* and *Bacteroidetes*, the predominant phyla in the gut microbiome of most mammals, have been closely correlated with energy partitioning and feed efficiency of pigs (Guo et al., 2008; Lu et al., 2018). Phylum *Bacteroidetes* has been shown to be more abundant in pigs with high feed efficiency or lean pigs compared to those with lower feed efficiency or obese pigs (Yan et al., 2016). Although no significant difference in the abundance of these two taxa was observed between M2 and FA groups in this study, *Firmicutes* and *Bacteroidetes* predominated in the fecal microbiome of all pigs, and the relative abundance of *Bacteroidetes* in the fecal microbiome of biphasic-fed pigs with high feed efficiency was slightly higher than that of pigs that had free access to feed. A previous study demonstrated that the gut microbiome of obese pigs harbored higher abundance of *Spirochaetes* than that of lean pigs (Yan et al., 2016). Analogously, the mutation of the myostatin-encoding gene has been shown to decrease the *Spirochaetes* abundance in the gut microbiome of pigs. In the present study, M2 pigs with high feed efficiency exhibited a lower abundance of *Spirochaetes* in feces compared to FA pigs. Although the carcass composition indexes were not measured in the present study, the biphasic feeding pattern has been indicated to ameliorate high-fat diet-induced fat deposition (Yan et al., 2020), implying that a higher *Spirochaetes* level might be associated with host adiposity. The *Euryarchaeota* is a phylum of archaea predominantly present in pigs at a late growth stage, of which the relative abundance was largely dependent on diet composition (Han et al., 2018). In this study, biphasic-fed pigs exhibited a lower abundance of *Euryarchaeota* in feces, which was inconsistent with the previous study, where *Euryarchaeota* was relatively higher in abundance in the high feed efficiency pigs (Quan et al., 2020). The contradictory findings might stem from the different dietary ingredient composition between the present study and the previous study. In the present study, the biphasic feeding regimen significantly decreased the abundance of genus *Subdoligranulum*, which has been positively correlated with lipid metabolic dysfunction and inflammatory response in pigs (Huang et al., 2020). In our previous study, the biphasic feeding pattern alleviated high fat-diet-induced lipid metabolic dysfunction and pro-inflammatory response in pigs (Yan et al., 2020), implying that genus *Subdoligranulum* might play an important role in this process. Genera *Roseburia* and *Mitsuokella* in the feces have been associated with increased growth and leanness in pigs (Gardiner et al., 2020). In the present study, the relative abundances of *Roseburia* and *Mitsuokella* were higher in feces of M2 pigs, which had higher feed efficiency. Additionally, biphasic-fed pigs had increased abundance of *Terrisporobacter*, which has been associated with better feed efficiency in young pigs (McCormack et al., 2019). The abovementioned studies supported the contention that the improvement effect of the biphasic feeding pattern on feed efficiency of pigs was associated with the altered fecal microbiome.

The perturbation of gut microbiota homeostasis would subsequently influence the intestinal metabolism (Alou et al., 2016), whereby we envisioned that biphasic feeding pattern-induced alterations in microbiota composition might be followed by the changes in the abundance of various metabolites in feces. In this study, a comprehensive analysis of the fecal metabolome of pigs fed for different regimens was implemented. The differentially enriched metabolites in the feces of M2 and FA pigs were identified, and most of these metabolites were accounted for amino acid catabolites and BAs. Functional analysis showed that the differential metabolites were involved in arginine, proline, glycine, serine, threonine, and tryptophan metabolism as well as primary BA biosynthesis. Genera *Subdoligranulum* and *Mitsuokella* have been positively correlated with fecal tryptophan metabolites in rodents and serum-free amino acids in pigs, respectively (Zhang D. et al., 2019; Zhang Z. et al., 2019). Moreover, genus *Roseburia* was known to be related to BA metabolism and body fat deposition (Kasahara et al., 2018). In the present study, genera *Subdoligranulum* and *Roseburia* were positively correlated with chenodeoxycholic acid and chenodeoxycholic acid 3-sulfate, which had the highest FC in abundance among all differential metabolites between FA and M2 pigs. A previous study showed that the changed gut microbiome and coupled altered stool BA profile mediated the protective effects of restricted time feeding on diet-induced obesity in mice (Chaix and Zarrinpar, 2015). Intermittent fasting has been shown to decrease obesity by enhancing the BA metabolism with higher primary and secondary BA levels in the gut lumen and serum (Li et al., 2017; Zhang et al., 2020). Therefore, we hypothesized that the perturbed microbiota-elicited changes in the BA profile would be responsible for the improvement effect that the biphasic feeding pattern had on feed efficiency of pigs. The increased concentrations of tauro-cholic acid, hyodeoxycholic acid, tauro-hyodeoxycholic acid, tauro-chenodeoxycholic acid, cholic acid, chenodeoxycholic acid, and deoxycholic acid in feces of biphasic-fed pigs could strongly support this hypothesis. These BAs are known as a potent agonist of the farnesoid X receptor (FXR), which plays a central role in modulating BA, lipid, cholesterol, and glucose metabolism (Calkin and Tontonoz, 2012). The activation of FXR by BAs induced the expression of intestinal fibroblast growth factor (FGF) 15/19, which has been shown to regulate skeletal muscle mass and ameliorate obesity-induced muscle atrophy (Benoit et al., 2017). Although these measurements were not determined in this study, intermittent fasting has been indicated to alleviate lipid metabolic dysfunction of diabetic mice associated with altered stool BA profile and the activation of the FXR-FGF15/19 pathway (Grant et al., 2017), implying that the altered BA profile in feces might contribute to the effect of the biphasic feeding pattern on feed efficiency of pigs.

## Conclusion

In conclusion, in terms of feed conversion efficiency of pigs, a biphasic feeding regimen could be better than a commonly adopted *ad libitum* regimen. The increased bacterial diversity, changed microbial composition, and altered fecal metabolome occurred in pigs fed biphasically. The altered fecal microbiota composition and enhanced stool BA pool could be responsible for the improvement effect of the biphasic feeding pattern on feed efficiency of growing–finishing pigs.

## Data Availability Statement

The datasets presented in this study can be found in online repositories. The names of the repository/repositories and accession number(s) can be found below: https://www.ncbi.nlm.nih.gov/, PRJNA756094.

## Ethics Statement

The animal study was reviewed and approved by the Institutional Animal Care and Use Committee of Southwest University of Science and Technology.

## Author Contributions

HY and JL designed and managed the project. WW, LH, and YZ analyzed the data, performed all animal works, and collected the biological samples. HY wrote the manuscript. HY, JL, and HZ revised the manuscript. All authors approved the final version of the manuscript.

## Conflict of Interest

The authors declare that the research was conducted in the absence of any commercial or financial relationships that could be construed as a potential conflict of interest.

## Publisher’s Note

All claims expressed in this article are solely those of the authors and do not necessarily represent those of their affiliated organizations, or those of the publisher, the editors and the reviewers. Any product that may be evaluated in this article, or claim that may be made by its manufacturer, is not guaranteed or endorsed by the publisher.

## Footnotes

1
http://www.metaboanalyst.ca
