# Supplementary material for: Reduced Feeding Frequency Improves Feed Efficiency Associated With Altered Fecal Microbiota and Bile Acid Composition in Pigs
Source: Front Microbiol. 2021 Oct 12;12:761210. doi: 10.3389/fmicb.2021.761210 (PMC8546368; doi:10.3389/fmicb.2021.761210)
Supplement: Supplementary file 1 [file Data_Sheet_1.PDF]

Supplementary Material

Table S1 The ingredient composition and nutrient level of diets

Composition (%)	Phase 1 (week 1-8)	Phase 2 (week 9-12)	Phase 3 (week 13-16)
Corn	68.50	69.90	73.00
Soybean meal (43% CP)	20.00	18.00	15.00
Wheat bran	7.00	5.00	5.00
Corn germ meal	0.00	3.00	3.00
Soybean oil	1.00	1.00	1.00
$CaHPO_4$	0.80	0.75	0.75
Limestone	0.66	0.52	0.47
NaCl	0.40	0.40	0.40
L-Lys·HCl	0.75	0.65	0.60
L-Trp	0.15	0.10	0.10
DL-Met	0.10	0.05	0.05
L-Thr	0.04	0.02	0.03
Choline chloride	0.10	0.10	0.10
Premix[1]	0.50	0.50	0.50
Total	100.00	100.00	100.00

[1]The premix provided the following per kg of the diet: Cu ($CuSO_4 \cdot 5H_2O$), 20 mg; Fe ($FeSO_4 \cdot 7H_2O$), 120 mg; Mn ($MnSO_4 \cdot H_2O$), 30 mg; Zn ($ZnSO_4 \cdot H_2O$), 120 mg; Se ($Na2SeO_3$), 0.5 mg; I (KI), 0.5 mg; Vitamin A, 8.000 IU; Vitamin D_3, 2.000 IU; Vitamin E, 12 IU; Vitamin K_3, 1.2 mg; Vitamin B_1, 1.5 mg; Vitamin B_2, 4 mg; Vitamin B_6, 2 mg; Vitamin B_{12}, 0.02 mg; biotin, 0.08 mg; pantothenic acid, 12 mg; nicotinic acid, 20 mg; folic acid, 0.5 mg.

Table S2 The dominant phyla (average abundance > 1.0%) in FA and M2 pigs

Treatment	Phylum	Average abundance
FA	*Firmicutes*	48.90%
	Bacteroidetes	41.65%
	Spirochaetes	4.87%
	Euryarchaeota	1.01%
M2	*Firmicutes*	46.71%
	Bacteroidetes	45.77%
	Spirochaetes	2.44%
	Proteobacteria	1.09%

FA, pigs had free access to feed; M2, pigs were given two meals per day (0800h and 1800h), each meal lasted 60 mins.

Table S3 The dominant genera (average abundance > 1.0%) in FA and M2 pigs

Treatment	Genus	Average abundance
FA	*unidentified_Spirochaetaceae*	1.05%
	Lactobacillus	3.14%
	unidentified_Prevotellaceae	3.40%
	Megasphaera	1.32%
	unidentified_Ruminococcaceae	2.78%
	Oscillospira	2.29%
	Alloprevotella	1.70%
M2	*Lactobacillus*	3.90%
	unidentified_Prevotellaceae	2.08%
	Streptococcus	1.09%
	Megasphaera	1.06%
	unidentified_Ruminococcaceae	2.57%
	Oscillospira	1.88%
	Alloprevotella	1.14%

FA, pigs had free access to feed; M2, pigs were given two meals per day (0800h and 1800h), each meal lasted 60 mins.

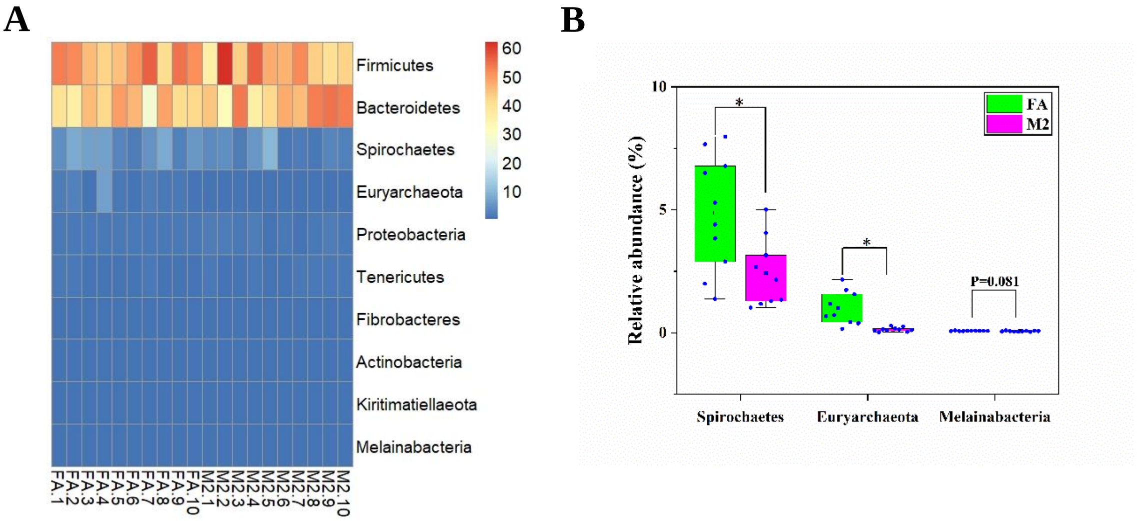

Figure S1. Phylum difference between FA and M2 groups. (A) Heat map of relative abundance of Phyla. (B) Significantly different phyla in feces between FA and M2 groups. FA, pigs had free access to feed; M2, pigs were given two meals per day (0800h and 1800h), each meal lasted 60 mins.

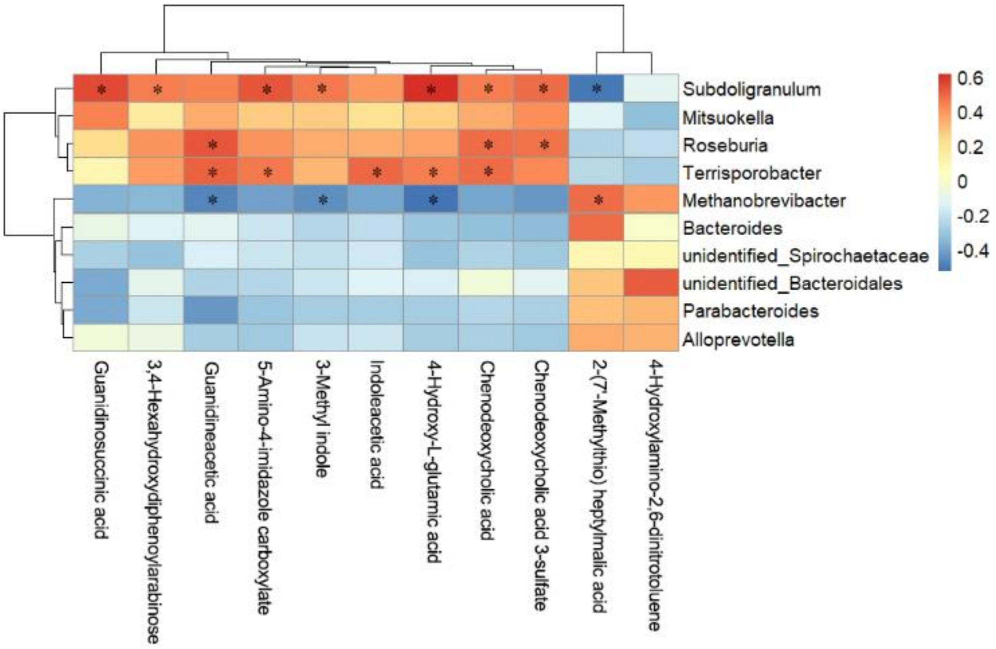

Figure S2. Spearman's correlation analysis between altered genera and metabolite concentrations in feces of pigs. Cells are according to Spearman's correlation coefficient between the significantly altered genera and metabolites. *P<0.05.
